# Subcutaneously implantable electromagnetic biosensor system for continuous glucose monitoring

**DOI:** 10.1038/s41598-022-22128-w

**Published:** 2022-10-17

**Authors:** Seongmun Kim, Jagannath Malik, Jong Mo Seo, Young Min Cho, Franklin Bien

**Affiliations:** 1 Department of Electrical Engineering, Ulsan National Institute of Science and Technology, 50, UNIST-Gil, Ulsan, 44919 Republic of Korea; 2 SB Solutions Inc., 29 Seoun-ro 6-gil, Seocho-gu, Seoul, 06731 Republic of Korea; 3 Department of Electrical and Computer Engineering, Seoul National University, Seoul, 151-742 Republic of Korea; 4 Department of Ophthalmology, College of Medicine, Seoul National University Hospital, Seoul, 110-744 Republic of Korea; 5 Department of Internal Medicine, Seoul National University College of Medicine, 101 Daehak-ro, Jongno-gu, Seoul, 03080 Republic of Korea; 6 Department of Internal Medicine, Seoul National University Hospital, Seoul, Republic of Korea

**Keywords:** Diagnostic markers, Biomedical engineering, Electrical and electronic engineering, Diabetes

## Abstract

Continuous glucose monitoring systems (CGMS) are becoming increasingly popular in diabetes management compared to conventional methods of self-blood glucose monitoring systems. They help understanding physiological responses towards nutrition intake, physical activities in everyday life and glucose control. CGMS available in market are of two types based on their working principle. Needle type systems with few weeks lifespan (e.g., enzyme-based *Freestyle Libre*) and implant type system (e.g., fluorescence-based *Senseonics*) with few months of lifespan are commercially available. An alternate to both working methods, herein, we propose electromagnetic-based sensor that can be subcutaneously implanted and capable of tracking minute changes in dielectric permittivity owing to changes in blood glucose level (BGL). Proof-of-concept of proposed electromagnetic-based implant sensor has been validated in intravenous glucose tolerance test (IVGTT) conducted on swine and beagle in a controlled environment. Sensor interface modules, mobile applications, and glucose mapping algorithms are also developed for continuous measurement in a freely moving beagle during oral glucose tolerance test (OGTT). The results of the short-term (1 h, IVGTT) and long-term (52 h, OGTT) test are summarized in this work. A close trend is observed between sensor frequency and BGL during GTT experiments on both animal species.

## Introduction

A growing research interest in healthcare systems has been observed in the last few decades, primarily focusing on the development of various point-of-care medical devices that can be of significant help to patients with financial or remote location constraints. Data base driven algorithms, multimodal information processing, and trend analysis are the key parts of bioinformatics that can help managing individual health, and issue predictive alerts to prevent fatal situations^1,2^. Metabolic diseases require the continuous management of eating habits, dietary plans, physical exercise, exposome and other general behaviors including sleep. The number of people with diabetes worldwide is 460 million as of 2019, and it is steadily increasing every year, with approximately 11.3% of deaths worldwide is due to the diabetes-related complications^3^. Diabetes mellitus increases further incidences of health complications exponentially^4,5^. The risk in diabetes can be minimized by monitoring and adapting habits to control blood glucose level (BGL). Commonly used method to measure BGL is using enzyme-based glucose sensor relying on electrochemical reaction^6,7^, known as amperometric sensor that produces measurable current proportional to BGL. This can be implemented in two ways either measuring directly from blood or from interstitial fluid (ISF). Former method is also known as self-blood glucose monitoring (SBGM) relies on finger-pricking method where a small blood is drawn from fingertip and put on glucose measuring strip. Although it measures glucose level with considerable accuracy, it is painful, causes skin irritation to the patients requiring frequent measurements. This method does not give any insight of BGL changes over time. Later method measures glucose level from the ISF through a needle inserted into the subcutaneous fat and connected to a measuring device placed and attached on skin. The device has main measurement unit and other control, communication units. This method is of one-time use, have short self-life (few days), expensive, and painful due to the insertion and presence of a needle for in-situ measurements^8–10^. It provides a BGL trend over continuous time, however the sensor life is few days. Extending the enzyme activity using electrochemical coating, sensor life could be increased from few days; however, limited to approximately two weeks of use^11,12^. Various methods alternate to enzyme-based blood glucose detection have been extensively reviewed to improve lifespan of sensor, accuracy of CGMS in literature^13,14^. Optical methods that use light-emitting diode (LEDs) of different wavelengths^15–17^ are also extensively studied for blood glucose detection. Usually, the sensor is a photo sensitive detector that can detect variations in optical intensity owing to variations in blood glucose level. Near-Infrared optical frequencies show glucose dependent intensity variations due to spectral absorption when illuminated, and the reflectance changes with the blood glucose variations. However, the strong absorption and weak intensity of the reflected signal affects the accuracy. To overcome this, improved optical methods using fluorescent materials with glucose selectivity have also been investigated^18,19^. The method uses a glucose-binding molecule that causes a change in fluorescent activity depending on the glucose level in blood. However, this method is limited by the degradation of the fluorescent material itself over time. As a result, the fluorescence reflectance diminishes gradually, and as time passes, it is barely detectable^20,21^.

The development of electromagnetic based glucose sensors has been studied in various lab experiments to non-invasive measurement of blood glucose level^22^. From a recent study it is understood that mechanism of glucose metabolism is not straightforward rather a sophisticated cascade of biochemical reactions. However, the dielectric response of water is directly affected by the glucose and a relevant marker for indirect measurement of glucose level in vivo^23^. The fundamental working principle of EM-based glucose sensors is to sense glucose dependent dielectric permittivity changes. The glucose dependent dielectric permittivity of blood has been characterized over a wide frequency range^24^. It has been observed that the dielectric permittivity decreases with increase in glucose level^25^. In general, glucose level dependent permittivity changes are reflected as a change in the resonance frequency of these EM sensors. Few of the relevant published works using electromagnetic based glucose sensor are in vitro measurement technique^26,27^ and wearable type measuring in vivo from outside body^28^. EM-based permittivity sensing biosensors have already proven to be effective for the detection of tumors and malignant cells in the body. The dielectric properties of various organs and tissues in living bodies have been previously characterized over a wide spectrum^29,30^. The resonance frequency of proposed EM-based implant sensor depends on permittivity of the surrounding environment. The sensor frequency changes inversely with changes in dielectric permittivity of the material in which sensor is embedded. The dielectric permittivity of blood changes as the glucose level changes. This glucose dependent permittivity changes are reflected in the sensor resonance frequency. Using a regression model, sensor frequency can be mapped to BGL. The EM-based sensors for BG measurement have already been attempted, and encouraging results have been reported in several research^31,32^.

EM resonators can be designed with different shapes and sizes and optimized for different frequencies of operation. Measurable parameters in the EM sensor that are indicative of the glucose level can be either reflection-based *S*_11_ or transmission-based *S*_21_ considering both magnitude and phase characteristics. Non-invasive measurement of glucose from outside the body has several challenges owing to high reflection of signals from the upper skin layer and low penetration depth of the signal^33^. The electromagnetic signal from the external transmitter experiences multiple reflections and strong attenuation from upper tissue layers. A marginal power that can reach subcutaneous layer and has weak sensing performance.

**Figure 1:**
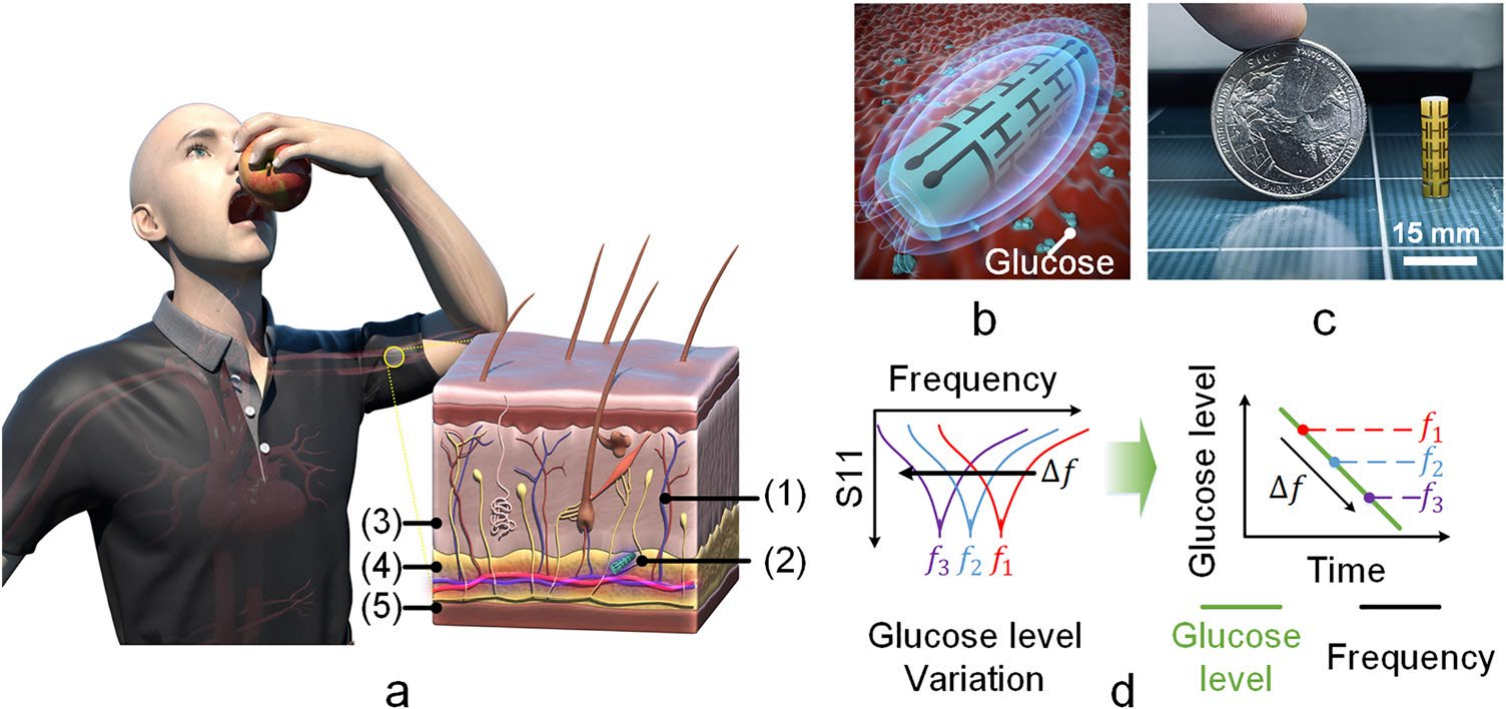
EM-based subcutaneous implant glucose sensor. (**a)** Illustration of EM-based implantable sensor for BGL tracking; (1) blood capillary (2) electromagnetic sensor (3) dermis (4) subcutaneous fat (5) muscle tissue. (**b)** Proposed implant sensor. (**c)** Sensor size (15 mm × 4 mm $\emptyset$) compared with a coin. (**d)** Sensor frequency trend and corresponding variations in BGL.

A minimally invasive implant type EM-based sensor is presented as a possible biosensor for real time BGL tracking. The proposed sensor is capable of detecting and tracking minute changes in the dielectric permittivity of interstitial glucose^34^ (Fig. 1a). The sensor was designed and simulated using full-wave electromagnetic simulator CST Microwave Studio. The sensor was optimized for maximum sensitivity considering a bio-environment similar to that of muscle and fat for ISM band. Here authors refer sensitivity as the frequency variations for small permittivity change of the material surrounding the sensor. The present sensor can’t detect glucose directly. The sensor frequency changes according to small permittivity changes. In the modelling of sensor in CST simulator, we consider permittivity variations of the material surrounding the sensor. Of course, we didn’t simulate the effect of glucose level on the permittivity of bio tissue environment, as it is not supported by the simulator. However, we did in vitro experiment to check effect of glucose level variations on permittivity of aqueous glucose solutions (supplementary file, method 1, Sect. 2).

The proposed implantable sensor is illustrated in Fig. 1b (also supplementary material Method 1, Sect. 1). The size of the sensor is compared against the size of a coin and shown in Fig. 1c. The sensor diameter is only 4 mm, compact enough and suitable for subcutaneous implantation. Figure 1d shows the sensor frequency variation with BGL trend. The sensor was subcutaneously inserted into the animal body. We performed both the intravenous glucose tolerance test (IVGTT) and oral glucose tolerance test (OGTT) with our sensor implanted in the animal (farm pig and beagle). In addition, we also developed a standalone sensor interface circuit board and mobile application that can continuously measure the sensor resonance frequency in the long-term evaluation of the sensor. Using the sensor system (EM sensor, interface circuit, and Android mobile application).

In the manuscript, short-term test is referred to one-time IVGTT test of the sensor performed on both swine and beagle, while long-term test is referred to a total 52 h (monitoring 17 h, measuring 35 h) OGTT experiment performed on a beagle. The long-term test on beagle was to verify continuous operation of sensor in tracking BGL while the animal was free to move around inside the experimental facility. Data processing algorithms such as linear regression and Kalman filter were used to remove fluctuations and high-frequency noise in the sensor reading. The mean absolute relative difference (MARD) and regression correlation coefficients were calculated to validate the sensor’s ability in tracking real time BGL.

## Results

### In vivo short-term IVGTT on a swine

In first in vivo experiment, we evaluated the sensor response towards real time blood glucose variations in a middle size farm pig. The sensor was subcutaneously implanted by the veterinary surgeon and IVGTT was conducted on the swine. At the implant site we observed body fluids, ISF and small trace of blood which was cleaned carefully before sensor implantation. After inserting the sensor and suture skin at the implant site, we continuously observed the sensor resonance frequency for approximately three hours before injecting glucose intravenously. We also injected phosphate-buffered saline (PBS) without any glucose and continuously recorded any change in the sensor resonance frequency. However, we did not observe any noticeable change in sensor resonance frequency. The total resonance frequency variations were significantly less than the frequency variation observed upon glucose injection.

**Figure 2:**
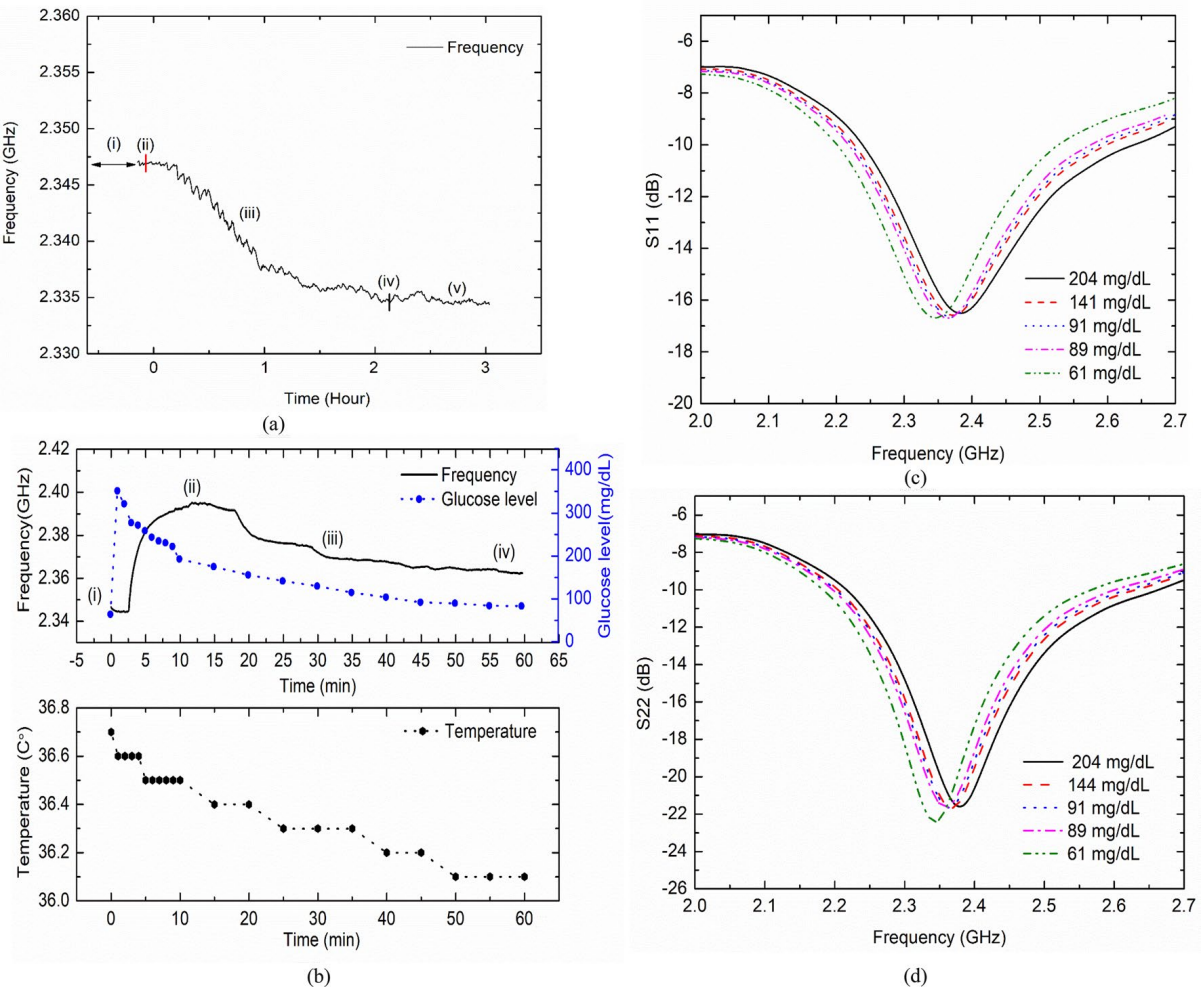
Results of the IVGTT conducted on swine. (**a)** Sensor insertion and initial frequency response filtered (i) surgery and sensor insertion (ii) monitoring (iii) frequency drifting (iv) saline (v) frequency drift saturate. (**b)** Sensor frequency response after glucose injection (i) Glucose solution injection, sensor resonance (ii) shifts to the maximum frequency, (iii) slowly decreases and follows the BG level, (iv) returns to the initial BG level, and body temperature variation during test. **(c)** $S_{11}$ parameter and (**d)** $S_{22}$ parameter with respect to BGL.

After sensor implantation, bio tissue surrounding sensor changes slowly and the adhesion with sensor improves over the time. This slow changes of tissue around sensor causes sensor frequency drift. However, after some time the process saturates/diminishes and sensor reference frequency is set. It should be noted that during short-term IVGTT experiment, the animal was given controlled anesthesia. Hence sudden body movement was not noticed which potentially disturbs sensor reference frequency. This was observed during OGTT experiment on freely moving beagle. Other factor such as animals has loose skin and can’t hold the sensor firmly at a location. This also disturbs the sensor reference frequency. Figure 1a shows the initial sensor resonance frequency behavior after surgery and insertion. We observed the continuous frequency drift to the lower side until 1 h and then slowly settling. We performed IVGTT by injecting glucose solution into the back leg vein. The sensor resonance frequency was recorded continuously with high sampling points setting in the vector network analyzer (VNA). We used two different methods, commercial blood glucose meter (BGM) and the standard Yellow Spring Instrument (YSI2500) to check BGL at every 5-min interval during IVGTT experiment. We observed an error margin within 10% between the two measured values with little higher reading on BGM at higher BGL ranges. The blood was drawn from the leg vein using a syringe. The blood sample was separated into blood plasma and red blood cell using a centrifuge as blood plasma have similar glucose level. The BGM (Caresens-N, i-SENS, Korea) reading were noted as the ‘*measured BG level’* and data readings from the YSI2500 were noted as the ‘*reference BG level’*. All glucose concentration was measured from the blood plasma. The BG level reached a maximum value at 376 mg/dL in a short period of time, and thereafter continued to decrease owing to natural insulin action of the body. The sensor resonance also followed a similar trend, albeit with a time delay. This is because the diffusion process of glucose from the blood vessel to ISF takes approximately 5–30 min depending on the metabolic rate of the living body^35^. A time delay of approximately 12 min was recorded between the peak BG level and the peak sensor resonance. The sensor frequency shifted by approximately 32 MHz, that is, from 2.395 GHz (the lowest BG level was 61 mg/dL) to 2.362 GHz (the highest BG level was 376 mg/dL) (Fig. 2b upper graph). The sensitivity is approximately 104 kHz/1 mg/dL (0.104 MHz/mg/dL) frequency variation from the peak BG variations. The body temperature of a swine was in the range of 36.4 ± 0.3 °C, which is similar to that of a human (Fig. 2b lower graph).

The proposed EM sensor resonance data (S-parameters, $$S_{11}$$ and $$S_{22} )$$ were continuously observed and recorded. Figure 2c,d show the resonance frequency point according to the BG level. The minima were tracked, and the trends are shown in Fig. 2b. The sensor resonance was observed around 2.4 GHz. The change in the resonance frequency was because of the change in the peripheral permittivity owing to the change in glucose level. As the glucose level increased, the resonant frequency shifted to a higher frequency band. The characteristics of $$S_{11}$$ and $$S_{22}$$ were slightly different but showed a similar trend.

### In vivo short-term IVGTT on a beagle

In the second experiment, short-term IVGTT was conducted on a healthy beagle to verify the sensor frequency sensitivity with glucose level changes. Here, the sensor-embedded bio-environment is different from the swine case. The beagle had a very thin subcutaneous layer compared to the swine. Therefore, it was difficult to implant the sensor in the subcutaneous fat. Hence, it was implanted between the muscle and the skin layer**.** In general, ISF is present and covers all tissue. Owing to the higher permittivity of muscle tissue compared to that of subcutaneous fat, the sensor after implantation in the beagle had a lower reference resonance frequency compared to swine case. Muscle has a higher dielectric permittivity (ε_r_ ~ 40) whereas subcutaneous fat (ε_r_ ~ 8). As a result, even with the same sensor, the reference resonance frequency moved about 300 MHz to the left in beagle compared to swine.

In addition, the temperature effect on the sensor behavior should not be neglected. Since, the dielectric permittivity is also affected by temperature variations. During experiment core body temperature of the animal subject measured using a rectal temperature sensor. Experiments were done inside a temperature-controlled surgery room. During first IVGTT experiment on swine, it was observed the body temperature of the swine dropped after surgery, anesthesia, and glucose injection. However, the sensor frequency was unaffected and followed the BGL trend. In the second IVGTT experiment on beagle, heating pads were used to keep animal warm and body temperature was maintain during experiment. The total variation in measured body temperature was 0.6 °C and 0.1 °C for swine and beagle respectively during experiment.

**Figure 3:**
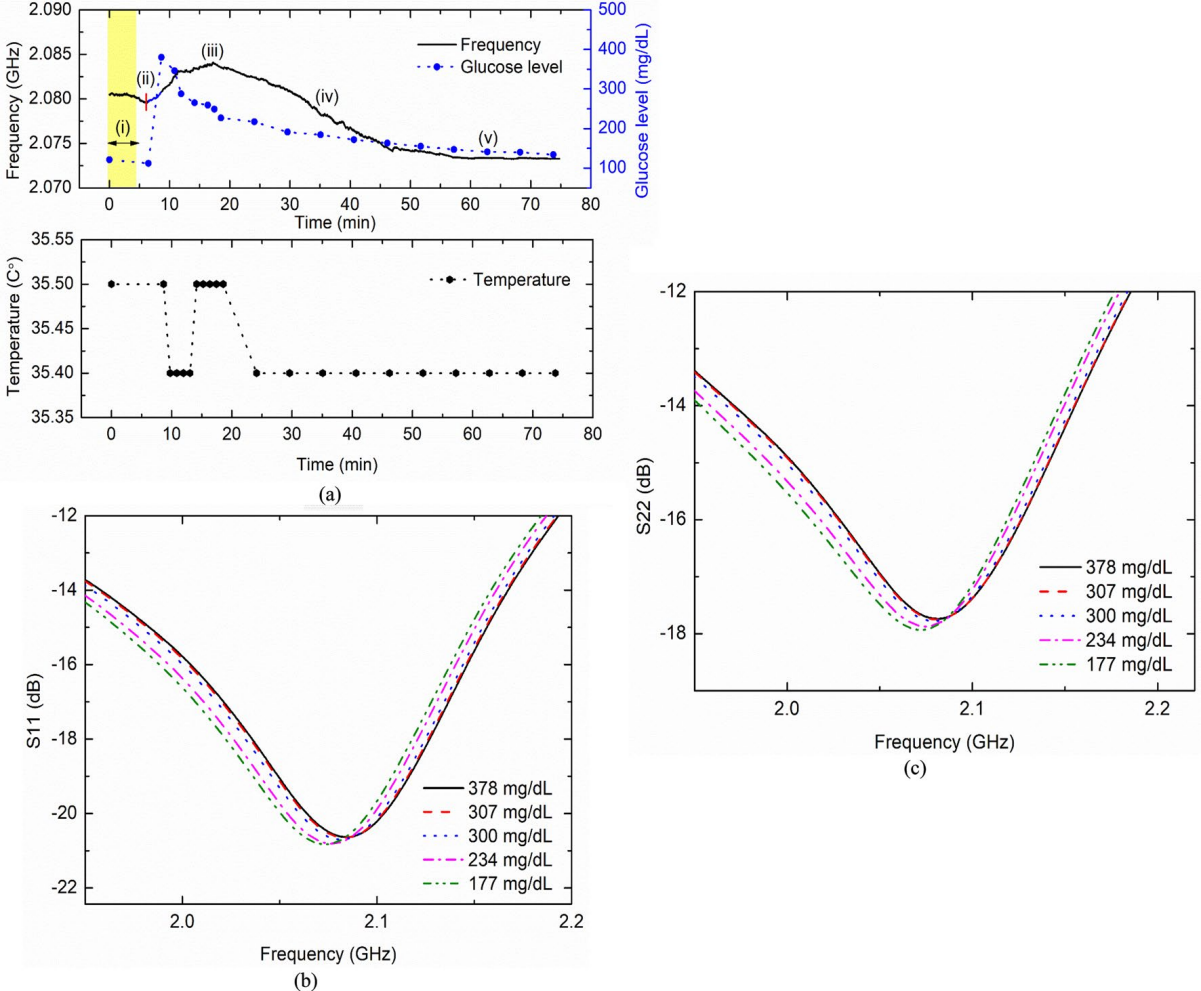
Results of the IVGTT conducted on a beagle. (**a)** Sensor resonance frequency variation with real-time BGL: Sensor frequency (i) before glucose injection (ii) after glucose injection (iii) maximum point (iv) decreasing and following BGL (v) does not vary as BGL does not vary, also shown body temperature monitoring, (**b)** $S_{11}$ and (**c)** $S_{22}$ parameter of the sensor and corresponding BGL.

After inserting the sensor into the beagle, the resonance frequency was measured continuously for approximately 2 h until the resonance frequency was reached at a stable condition without variation. Thereafter, PBS without glucose was administered into the beagle and the sensor frequency was continuously observed (1 h). This was done to mimic the sham test and verify the sensor behavior. Then, the IVGTT was performed, and continuous sensor resonance measurement and BG level measurement at regular 5-min time intervals were performed. As BG changed, the sensor frequency also changed. The sensor frequency and trend in beagles varied in a similar way as in the case of swine. As soon as the glucose solution was injected, the BG level rapidly increased compared to the sensor frequency (Fig. 3a upper). The delay between the BG level and sensor frequency was approximately 10 min due to the glucose diffusion process from the blood to the ISF.

When the BG level changed from 450 to 129 mg/dL (Δ*Gl* = 321 mg/dL), the resonant frequency shifted from 2.0836 GHz to 2.0732 GHz (Δ*f* = 10.34 MHz). As a result, the sensitivity change with respect to BG was approximately 32 kHz *per* 1 mg/dL of change in BG levels (Δ*f*/Δ*Gl* = 10.34/321 MHz/mg/dL). The variations in rectal temperature were recorded using a precision thermistor sensor inserted into the body. The temperature change was 35.4–35.5 °C during the experiment (Fig. 3a lower). With an increase in the BG level, the resonance frequency also increased. When the BG level decreased, the resonance frequency shifted to a lower frequency (Fig. 3b,c).

### In vivo long-term OGTT on a beagle

**Figure 4:**
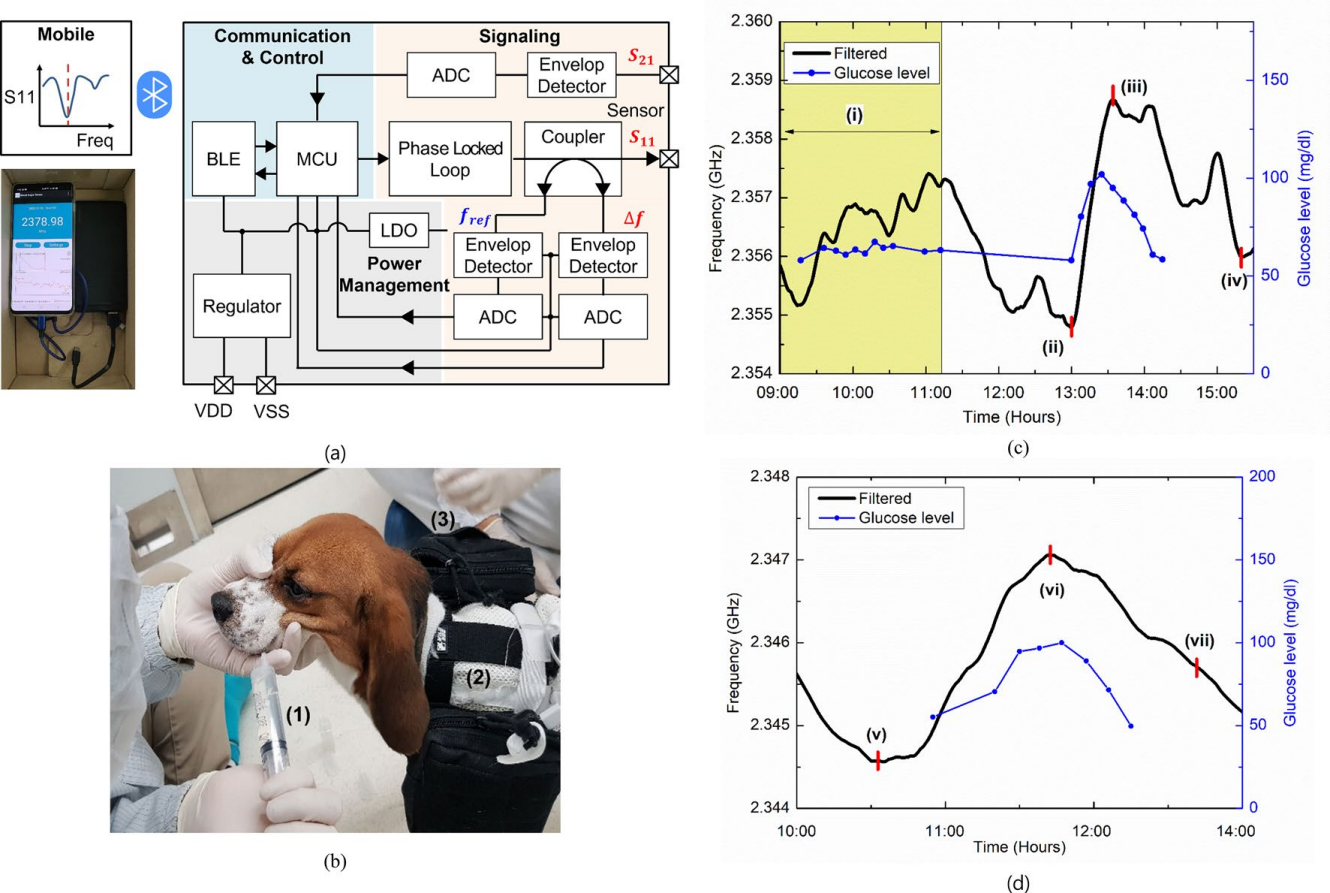
OGTT experiment on beagle using sensor and interface board. (**a)** Proposed sensor and interface circuit board (Android app receive the sensor information using Bluetooth, MCU controller, PLL for RF generation and input to sensor); Interface board consists of three major parts: power management part (voltage regulator, LDO); RF part (PLL, wideband coupler, envelope detector); digital part (ADC, MCU). (**b**) OGTT (beagle fed with glucose solution): (1) Glucose solutions (2) Interface board (3) Battery. (**c**) Day-1 OGTT: (i) Trend of BGL and sensor frequency while feeding the beagle; (ii) first oral glucose administration; (iii) maximum BGL and corresponding sensor frequency with a time lag; (iv) sensor frequency decreases as the BGL decreases. (**d**) Day-2 OGTT (v) Second oral glucose administration; (vi) maximum BGL and corresponding sensor frequency with a time lag; (vii) sensor frequency decreases as the BGL decreases.

After the initial in vivo IVGTT experiments were performed on swine and beagle, sensor resonance frequency and operational bandwidth were analyzed for the development of sensor interface circuit board. During IVGTT the animals were anesthetized, and network analyzer (E5071C, Keysight) attached with long RF cables was used to measure sensor frequency. However, this setup was not suitable for conducting long-term tests as the animal was not under anesthesia. Moreover, it was dangerous to keep the animal under anesthetized for a long time. Therefore, the sensor was implanted, and connected to the interface board for continuous monitoring. The portable glucose monitoring system consisted of a sensor and an interface board (Fig. 4a). The sensor was inserted under the skin, and the interface board was taped outside the body. To supply power to the sensor and interface board, a high-capacity battery was also attached on a supporting jacket. The jacket was used to firmly hold the battery such that the sensor connections are not affected by movements of the beagle. The interface board can continuously measure the sensor resonance frequency without any interruption and send the data to the Android mobile application through a Bluetooth link (supplementary materials method 2, Sect. 4). After implanting the sensor, the beagle was kept inside a cage and allowed to move freely outside the cage. The animals were kept overnight in normal conditions and fed with food and water until the next morning. During this period, no glucose injection or BG level monitoring was performed. The sensor frequency BG level was measured in the morning of the next day with OGTT (Fig. 4b). The measurement plot is divided in four different zones (Fig. 4c). ‘Zone (i)’ shows the measurement results when the beagle was fed and ‘Zone (ii)’ shows the oral glucose feeding and corresponding sensor frequency variation. A small change in BG and a similar change in the sensor frequency can be seen. Instead of injecting glucose solution intravenously, it was administered orally. The oral glucose administration during OGTT did not increase the BG level in the beagle as fast as IVGTT. This is because glucose spreads to the blood through the digestive tract, and then it spreads from blood to ISF, which is a slower process. After reaching the highest point ‘Zone (iii), the sensor frequency also decreased following the BG level ‘Zone (iv)’. Strong fluctuations in the sensor reference frequency were observed due to the body movements of the beagle. The results of the second OGTT also showed a similar trend as shown in Fig. 4d. The increasing sensor frequency response with BGL is shown in ‘Zone (v)’. During the OGTT experiment, a similar trend of increasing sensor frequency to the peak ‘Zone (vi)’ and then decreasing with BGL ‘Zone (vii)’ were observed.

### Frequency to BGL regression and analysis

**Figure 5:**
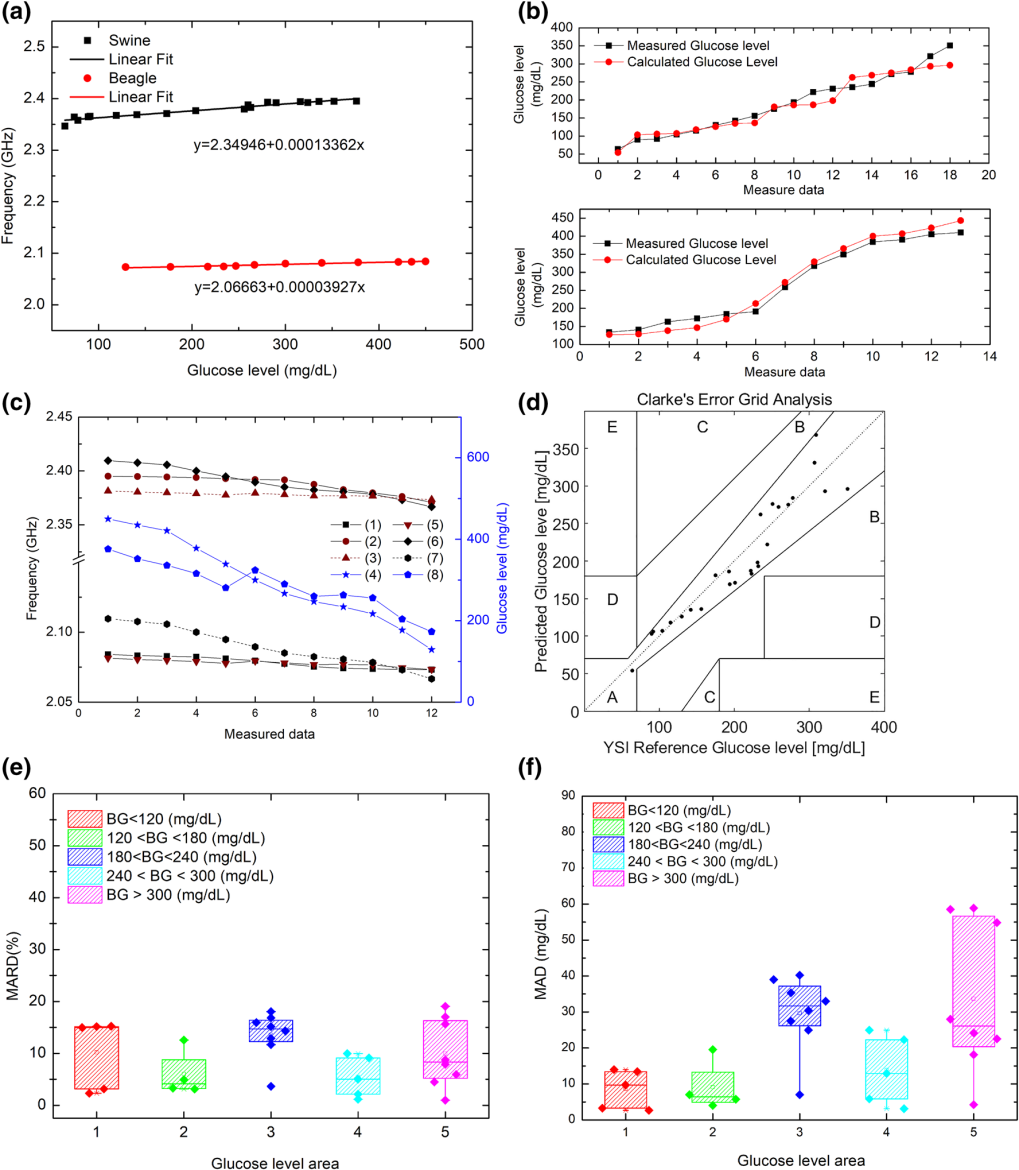
analysis results of Linear Regression, MARD, MAD, and EGA. (**a**) Analysis of linear regression about swine ($R^{2} = 0.92604$) and beagle ($R^{2} = 0.93399$). (**b**) Compared between calculated BGL and reference BGL: (1) swine (upper) (2) Beagle (lower). (**c**) using regression equation of swine on beagle BGL measurement data and vice versa: (1) beagle measurement, (2) Swine measurement (3) Swine data offset (4) Beagle Glucose level (5) Swine Calculation (6) Beagle calculation (7) Beagle data offset (8) Swine Glucose level. (**d**) EGA for the beagle and swine (Zone-A: 92.31% n = 24, Zone-B: 7.69% n = 2, Zones-C, D, E: 0%). (**e**) Change in MARD values according to the glucose level areas (n = 30). (**f**) Comparison between reference BGL and predicted BGL. The distribution (n = 30) was determined by dividing the glucose level into five glucose level areas. (1) The average value of MAD was 8.6119 in the standard BGL range (BG < 120 mg/dL, n = 5). (2) The average MAD value was 9.0977 for the glucose level area of pre-diabetes and diabetes (120 < BG < 180 mg/dL, n = 4). (3) The average MAD value was 17.2447 in the glucose level area of diabetes (180 < BG < 240 mg/dL, n = 8). (4) The average MAD values of the high-risk and hyperglycemia groups (240 < BG < 300 mg/dL, n = 5 BG > 300 mg/dL, n = 8) were 12.6545 and 33.6701 mg/dL, respectively.

During all in vivo IVGTT and OGTT experiments, after intravenous glucose administration or oral glucose administration, the BGL was observed to change. It moved higher, reached peak level then decreased naturally. The sensor frequency in each case also followed the BGL trend. The proposed sensor can track the BGL trend from a reference point. Sensor frequency to BGL mapping can be derived for calculating BGL for certain sensor frequency. The linear regression and correlation between the BGL and sensor frequency are derived and shown in Fig. 5a. the black line is for swine linear fit and the red line is for beagle linear fit. The proposed sensor shows good linearity with BGL in both cases. Even though two modeling linear regression are not same for different animal, the proposed sensor worked in a similar manner. The y-intercept of the regression is the reference frequency of sensor in individual cases. It can be seen that there is an offset in reference frequencies in both cases. Figure 5b was difference glucose level between reference measurement and calculation by each linear regression arranged from lower to higher BGL. The calibration coefficients of the linear models for the beagle and the pig are similar but not the same. For the generalized performance of the sensor, we inverse calculate sensor frequency for swine using beagle calibration equation with beagle BGL data, and vice versa. Since the sensor reference frequency in case of swine and beagle are different, an offset in frequency was added. From Fig. 5c it can be seen that original calibration line and inverse calculated frequency has similar trend, however a higher difference is observed for higher BGL values. This is mainly limited availability of BGL data points to derive original calibration line. This can be improved with more experiments and more data collection. The Clarke’s error gird analysis (EGA) is shown in Fig. 5d. It can be seen that data points are mostly distributed in Zone-A and Zone-B. The accuracy of each BG level range was ascertained using the MARD value. Accuracy could be checked through error grid analysis (EGA); however, it was difficult to determine the range in which the accuracy was high and low due to availability of fewer reference blood glucose data. Therefore, the MARD values were classified and analyzed according to the ranges. MARD results are separated into different zones as shown in Fig. 5e and supplementary material method 3 Sect. 7. The mean absolute deviation (MAD) results (Fig. 5f) were separated in five ranges according to BG levels. In the Range 1 i.e., the normal range (BGL < 120 mg/dL), the reference and error values had a small difference of 8.6119 mg/dL. In the Range 2 is defined as the area above normal glucose level (20 mg/dL < BGL < 180 mg/dL). The relative average error was found to be 9.0977 mg/dL. Even though the minimum and maximum error values increased compared to the results of Range 1, the overall error it is still within the margin. Nevertheless, the sensor had a high linearity of BG and frequency change. When the reference glucose level and the measured glucose value were compared, the average value for MAD was calculated to be 18.94 mg/dL. MARD, which is the most important index in BG measurement, was within 9.67% in Table 1.

**Table 1:** IVGTT result summary.

Parameters	Beagle	Swine
Linear regression (Y: Freq. X: BGL)	Y = 2.06663 + 0.00003927x	Y = 2.34946 + 0.00013362x
$R^{2}$ fit	0.93399	0.92604
BGL range (mg/dL)	129–450	64–376
Frequency change (Max. to Min)	$10$ MHz	$32$ MHz
Sensitivity *per* 1 mg/dL	42 kHz	112 kHz
Average (MAD)	18.94 mg/dL	
MARD	9.67%	

## Conclusion

Present work is an effort for the realization of implantable electromagnetic-based sensor, which can be an alternate to enzyme-based or optical-based glucose sensor. The sensor does not detect or track glucose molecules directly from blood or ISF, rather the resonance frequency of the sensor changes with changes in dielectric permittivity of ISF owing to change in BGL. In a way, it can track blood glucose trend from a reference or calibration point. Once a day calibration with SBGM can be used to measure blood glucose and set the reference frequency point of sensor. A linear regression model between sensor frequency and BGL is also developed for frequency to BGL mapping. Initial proof-of-concept in vivo experiment was performed with sensor implanted to swine and beagle in a controlled environment. A good correlation between sensor frequency and BGL is seen during in vivo IVGTT and OGTT experiments on swine and beagle. In addition, the developed sensor interface module is capable of continuous measurement, and the real-time sensor data can be visualized using the Android mobile application. Our proposed sensor and system are indeed in the early stage of development. However, the proof-of-concept in vivo results show promising correlation between BGL and sensor frequency response. Indeed, the sensor shows the ability to track BGL trend. For actual sensor implantation we must consider bio compatible packaging and foreign body reactions (FBR) for long term applications. In addition, improved sensor interface system is under development.

## Experimental methods

The study and experimental procedure were carried out in accordance with the ARRIVE guidelines. All experiments were performed according the relevant guidelines and recommendations. Experiments were performed at Animal Testing Center of KBIO Health Medical Device Development Center (Osong Medical Innovation Foundation, Osong, Chungbuk, Korea) following approval of the Institutional Animal Care and Use Committee (KBIO-IACUC-2020-172).

We performed animal experiment for in vivo evaluation of our sensor on swine (n = 1, Cornex, Korea) and beagle (n = 1, Orient bio, Korea). The experiment was conducted on healthy animals after the acclimation period of 1 week. For the short-term test, anesthesia was done by the veterinarian. Intramuscular injection of tiletamine-zolazepam 5 mg/kg (Zoletil®, virbac, south korea) and Xylazin 2 mg/kg (Rompun®, bayer, south korea) at endotracheal tube intubation was performed. Anesthesia was maintained with 1–1.5% isoflurane (Terrell™, piramal critical care, USA) using anesthesia machine (Fabius GS premium, dragger, Germany) during the experiment.

### Biosensor based on an EM resonator

Depending on their far- and near-field characteristics, EM resonators are widely used as communication antennas and sensors, respectively. A stronger far field leads to a radiative nature with excellent transmission and reception in wireless communication systems. However, they are not suitable for sensing applications. The EM property near the field is highly suitable for sensing and detecting applications ranging from EM sensors to actuators. The proposed sensor utilizes high-quality (ratio of stored energy to radiated energy) resonance with a strong oscillating near field. As an inherent interaction mechanism, EM-based sensors can be designed to be significantly sensitive in detecting dielectric permittivity changes. Suitably designing an EM resonator with tailored near-field energy confinement in a thinner structure (compared to the guided wavelength) is the key behind these sensors. This minimized the radiative energy from the sensor.

Metamaterials have been the most popular over the past few decades for designing electromagnetic resonators and structures for various applications. The planar (2D) versions of these materials are called metasurface (MTS). These MTS are periodic in nature by repeating a structure called a unit cell. The EM properties of the MTS are controlled by suitably designing the unit cell. The proposed sensor has these advantages and can be realized using a truncated MTS. The regular periodic nature of metamaterials limits their benefits in realizing compact sensors. However, in the present case, a special two-port coupled excitation was designed and tuned to achieve resonance in a compact truncated MTS. The sensor is a combination of an MTS unit cell excited with coupled feed line excitation. The coupled feed line was chosen because of its coplanar structure, with one side requiring metallization (copper) suitable for our case (supplementary material Method 1, Sect. 1, 2 and 3).

### Sensor interface module for long-term measurement

The scattering parameters are analyzed to completely characterize the sensor behavior toward changes in BGL. Network analyzer was used to measure the sensor resonance characteristics during the short-term test. However, bulky VNA and long RF cables are not suitable for long-term measurements. Therefore, a compact sensor interface module powered by a high-capacity battery was used for long-term measurements. The implant glucose sensing system consists of three functions: power management, RF section, communication, and control, which can communicate data to the outside by using Bluetooth function (supplementary material Method 2, Sect. 4).

### Short-term IVGTT for in-vivo studies of a swine

The first in vivo test was conducted on a swine. It had a similar BG variation to that of humans. Therefore, it was reasonable to implant our sensor and perform an IVGTT to evaluate the sensor behavior. The swine used was a farm swine (Cornex, Republic of Korea). The weight of the four-month-old female swine was approximately 40 kg. The body temperature was 37.2 °C. The swine was under fasting conditions for 24 h before the experiment. The sensor was inserted into the subcutaneous fat of the back flank. The subcutaneous layer of swine was considerably thick compared to other animals and the area was easily distinguished between muscle layer and subcutaneous fat layer to insert the sensor. Bleeding was observed when the skin was incised at the sensor implantation site. A heated knife was used to incise the skin to avoid local bleeding. Small blood traces were carefully removed and wiped as it interferes with sensor characteristics A heating pad (Blanketrol-II, Gentherm, USA) was used to maintain the subject's body temperature during the insertion procedure and experiments. After inserting the sensor into the body of the subject, an IVGTT was conducted^36^. A 20% glucose solution was injected through the ear vein. A commercial blood glucose meter and YSI2500 were used to measure BGL from blood sampled from leg vein at regular intervals. Sensor frequency and measured BGL was used for frequency to BGL mapping.

### Short- and long-term GTTs for in-vivo studies of a beagle

In biomedical research, beagles are usually considered for proof-of-concept evaluation to device performance measurements during development of various biomedical sensors. It is a suitable candidate to us for the short-term measurements as well as long-term measurements of proposed sensor^37^. The beagle was 22-months old female weighing 12.5 kg. The sensor was inserted under the skin and connected to a network analyzer for measurements in short-term experiment. The change in the sensor resonance frequency and corresponding BG level were recorded. Whereas, for long-term measurements we used a customized compact measurement circuit board that detects sensor frequency and transmits the data to a mobile using Bluetooth link. An Android app running on the mobile displays the information in a graphical view.

The IVGTT was conducted by injecting a 20% glucose solution into the leg vein, and blood was collected from the opposite leg. In the case of long-term measurements, the subject cannot be put under anesthesia for a long time; hence, a sensor interface board was developed. Long term test’s experimentally time was totally 52 h (monitoring 17 h, measuring 35 h). After surgery to the implant sensor, the subject (animal) needed self-curing time to be normalized about body condition such as maintaining normal temperature, stopping bleeding etc. The sensor interface board is compact and can be programmed to track the sensor’s resonance point. Blood was collected for a certain period to ensure the glucose trend. In the long-term experiment, 20% glucose solution was used in the OGTT.

### Data processing

The data were recorded continuously during the short term IVGTT experiments using a vector network analyzer and during long-term sensor measurements using in-house developed sensor interface circuit board. We observed more noise in the measured frequency data during the long-term experiment. Firstly, the sudden changes in the frequency are motion induced spikes due to the movement of beagle. Another source of noise was introduced by the measurement circuit itself due to ADC (analog to digital conversion) resolution. Since, we used a network analyzer to measure sensor resonance frequency during short-term experiment, the data were much cleaner compared to the long-term measurement. We used moving averaging filter to remove sudden high changes and fluctuations in measured frequency data. Depending on the window size, the memory requirement also changes. A larger window size requires more memory to save previous time series data, but gives a smother filtering compared to that of smaller window size filter. A larger window may also remove small and valid trends in data. It smoothens the frequency trend, however also introduce time lag depending on the filtering window size (supplementary materials Fig. S5c). A larger averaging window also requires more storage memory. An alternate method is to use Kalman filter which requires only last time step data to calculate/ predict filtered data. In case of long-term test, it can be seen that it has sudden and high rate of change in frequency. So, we applied Kalman filtering to discard those spikes. The noise in measured data was also due to various factors e.g., motion induced fluctuations (due to movement of beagle), body fluid accumulation around sensor, and changes in tissue contact surrounding the sensor over time. Owing to these factors, the resonance frequency was not clean compared to the situation when the sensor was suspended in air.

Considering time required for each full range frequency sweep and wireless data (measured sensor frequency information) transmission, 12 times *per* minute sample were possible and recorded. A higher sampling rate over a narrow frequency span around sensor resonance point can reduce noise significantly. However, the memory requirement and processing time was a limiting factor. The Kalman filter was applied to find a clear trend in a noisy measurement environment^38^ (supplementary material Method 3, Sect. 5). It clearly removes high-frequency fluctuations compared with a regular averaging filter. Linear regression modeling was also performed on the filtered data to calculate the correlation between sensor frequency and BG level^39^ (supplementary material Method 3, Sect. 6). Glucose level corresponding to a sensor frequency can be calculated (predicted) using the linear regression equation. The MAD (Mean absolute deviation) MARD (Mean absolute relative difference) was calculated by comparing the predicted BG level with the reference BG level. The MAD and MARD was defined in Eqs. (1) and (2).

1 $${\text{MAD}} = \frac{1}{{\text{n}}}\mathop \sum \limits_{{{\text{i}} = 1}}^{{\text{n}}} \left| {{\text{BG}}_{{{\text{predicted}}}} \left( {\text{i}} \right) - {\text{BG}}_{{{\text{Ref}}}} \left( {\text{i}} \right)} \right|$$

2 $${\text{MARD}} = \frac{1}{{\text{n}}}\mathop \sum \limits_{{{\text{i}} = 1}}^{{\text{n}}} \left| {\frac{{{\text{BG}}_{{{\text{predicted}}}} \left( {\text{i}} \right) - {\text{BG}}_{{{\text{Ref}}}} \left( {\text{i}} \right)}}{{{\text{BG}}_{{{\text{Ref}}}} \left( {\text{i}} \right)}}} \right|$$

${\text{BG}}_{{{\text{predicted}}\left( {\text{i}} \right)}}$ was the measured glucose concentration value that was converted from frequency to glucose level. That value was derived through linear regression. ${\text{BG}}_{{{\text{Ref}}\left( {\text{i}} \right)}}$ was measured using YSI2500).${\text{ n}}$ represents the number of measurements. MAD indicated error value between ${\text{BG}}_{{{\text{predicted}}\left( {\text{i}} \right)}}$ and ${\text{BG}}_{{{\text{Ref}}\left( {\text{i}} \right)}}$. MARD was an indication of error rate scale between predicted glucose level and reference glucose level. Generally, a BG meter has an accuracy of 15% MARD^40^ (supplementary material Method 3, Sect. 7). From the EGA analysis, it can be seen that the sensor data are uniformly distributed from the hypoglycemic to the hyperglycemic range^41^. This ensures the sensor is capable of tracking BGL variations with high confidence.

## Supplementary Information

Supplementary Information.

## Data Availability

We declare, datasets generated and/or analyzed during the current study are not publicly available due to regulations from the funding agency. However, it can be made available from the corresponding author upon reasonable request.

## References

[1] Hosseini, M. P., Lau, A., Elisevich, K. & Soltanian-Zadeh, H. Multimodal analysis in biomedicine. *Big Data Multimodal Med. Imaging *193 (2019).

[2] Behrad, F. & Abadeh, M. S. An overview of deep learning methods for multimodal medical data mining. *Expert Syst. Appl.* 117006 (2022).

[3] International Diabetes Federation 9th Edition. International Diabetes Federation (2019).

[4] Cui Y (2017). Prevalence and causes of low vision and blindness in a Chinese population with type 2 diabetes: The Dongguan Eye Study. Sci. Rep..

[5] Fox CS (2015). Update on prevention of cardiovascular disease in adults with type 2 diabetes mellitus in light of recent evidence: A scientific statement from the American Heart Association and the American Diabetes Association. Circulation.

[6] Lee H, Hong YJ, Baik S, Hyeon T, Kim DH (2018). Enzyme-based glucose sensor: From invasive to wearable device. Adv. Healthcare Mater..

[7] Wang J (2008). Electrochemical glucose biosensors. Chem. Rev..

[8] Klonoff DC (2005). Continuous glucose monitoring: Roadmap for 21st century diabetes therapy. Diabetes Care.

[9] Yoo E-H, Lee S-Y (2010). Glucose biosensors: An overview of use in clinical practice. Sensors.

[10] Nichols SP, Koh A, Storm WL, Shin JH, Schoenfisch MH (2013). Biocompatible materials for continuous glucose monitoring devices. Chem. Rev..

[11] Yu Z, Jiang N, Kazarian SG, Tasoglu S, Yetisen AK (2021). Optical sensors for continuous glucose monitoring. Prog. Biomed. Eng..

[12] Bobrowski T, Schuhmann W (2018). Long-term implantable glucose biosensors. Curr. Opin. Electrochem..

[13] Lee I, Probst D, Klonoff D, Sode K (2021). Continuous glucose monitoring systems-Current status and future perspectives of the flagship technologies in biosensor research. Biosens. Bioelectron..

[14] Didyuk O, Econom N, Guardia A, Livingston K, Klueh U (2021). Continuous glucose monitoring devices: Past, present, and future focus on the history and evolution of technological innovation. J. Diabetes Sci. Technol..

[15] Yetisen AK (2017). Glucose-sensitive hydrogel optical fibers functionalized with phenylboronic acid. Adv. Mater..

[16] Mohammadi LB (2014). In vivo evaluation of a chip based near infrared sensor for continuous glucose monitoring. Biosens. Bioelectron..

[17] Kang JW (2020). Direct observation of glucose fingerprint using in vivo Raman spectroscopy. Sci. Adv..

[18] Pickup JC, Hussain F, Evans ND, Rolinski OJ, Birch DJ (2005). Fluorescence-based glucose sensors. Biosens. Bioelectron..

[19] Kim Y, Jang G, Kim D, Kim J, Lee TS (2016). Fluorescence sensing of glucose using glucose oxidase incorporated into a fluorophore-containing PNIPAM hydrogel. Polym. Chem..

[20] Sawayama J, Okitsu T, Nakamata A, Kawahara Y, Takeuchi S (2020). HHydrogel glucose sensor with in vivo stable fluorescence intensity relying on antioxidant enzymes for continuous glucose monitoring. Science.

[21] Vaddiraju S, Burgess DJ, Tomazos I, Jain FC, Papadimitrakopoulos F (2010). Technologies for continuous glucose monitoring current problems and future promises. J. Diabetes Sci. Technol..

[22] Saha S (2017). A glucose sensing system based on transmission measurements at millimetre waves using micro strip patch antennas. Sci. Rep..

[23] Caduff A, Ben Ishai P, Feldman Y (2019). Continuous noninvasive glucose monitoring; water as a relevant marker of glucose uptake in vivo. Biophys. Rev..

[24] Omer AE, Safavi-Naeini S, Hughson R, Shaker G (2020). Blood glucose level monitoring using an FMCW millimeter-wave radar sensor. Remote Sens..

[25] Topsakal, E., Karacolak, T. & Moreland, E. C. Glucose-dependent dielectric properties of blood plasma. In *2011 XXXth URSI General assembly and Scientific Symposium*, 1–4 (2011).

[26] Hofmann M, Fischer G, Weigel R, Kissinger D (2013). Microwave-based noninvasive concentration measurements for biomedical applications. IEEE Trans. Microw. Theory Tech..

[27] Tang L, Chang SJ, Chen CJ, Liu JT (2020). Non-invasive blood glucose monitoring technology: A review. Sensors.

[28] Hanna J (2020). Noninvasive, wearable, and tunable electromagnetic multi sensing system for continuous glucose monitoring, mimicking vasculature anatomy. Sci. Adv..

[29] Gabriel S, Lau RW, Gabriel C (1996). The dielectric properties of biological tissues: III: Parametric models for the dielectric spectrum of tissues. Phys. Med. Biol..

[30] Ibrani M, Ahma L, Hamiti E (2012). The age-dependence of microwave dielectric parameters of biological tissues. Microw. Mater. Char..

[31] KIm Nam-Young, Adhikari ZKishor Kumar, Dhakal Rajendra, Chuluunbaatar Zorigt, Wang Cong, Kim Eun-Soo (2015). Non-invasive continuous-time glucose monitoring system using a chipless printable sensor based on split ring microwave resonators. Sci. Rep..

[32] Baghelani M, Abbasi Z, Daneshmand M, Light PE (2020). Non-invasive continuous-time glucose monitoring system using a chipless printable sensor based on split ring microwave resonators. Sci. Rep..

[33] Willemijn Groenendaal M.Sc., Basum Golo Von, Schmidt Kristiane A, Hilbers Peter A. J., Van Riel Natal A. W. (2010). Quantifying the composition of human skin for glucose snesor development. J. Diabetes Sci. Technol..

[34] Eda Cengiz, Tamborlane William V (2009). A tale of two compartments interstitial versus blood glucose monitoring. Diabetes Technol. Ther..

[35] Tomas Koutny (2014). Blood glucos level reconstruction as a function of A tale of Two Compartments transport. Biol. Med..

[36] KANEKO J.J, MATTHEEUWS D, ROTTIERS R.P., VERMEULEN A. (1978). Glucose tolerance and insulin response in diabetes mellitus of dogs. J. Small Anim. Pract..

[37] Manell Elin, Hedenqvist P, Svensson A, Jensen-Waern M (2016). Establishment of a refined oral glucose tolerance test in pigs, and assessment of insulin, glucagon and glucagon-like peptide-1 responses. PLoS ONE.

[38] Rebrin K, Sheppard N.F.Jr., Steil G.M. (2010). Use of subcutaneous interstitial fluid glucose to estimate blood glucose: revisiting delay and sensor offset. J. Diabetes Sci.Technol..

[39] Dorsaf G., Yassine M, Khaled N (2019). Comparison of linear and non-linear regression models for non-invasive blood glucose measurement. J. Comput. Sci.

[40] Anand P.K., Shin D.R., Memon M.L. (2020). Adaptive boosting based personalized glucose monitoring system (PGMS) for non-invasive blood glucose prediction with improved accuracy. Diagnostics.

[41] Clarke, W. L. The original Clarke error grid analysis (EGA). *Diabetes Technol. Ther.* **7**(5), 776–779 (2005). 10.1089/dia.2005.7.776 16241881

