# Supplementary material for: Subcutaneously implantable electromagnetic biosensor system for continuous glucose monitoring
Source: Sci Rep. 2022 Oct 17;12:17395. doi: 10.1038/s41598-022-22128-w (PMC9576697; doi:10.1038/s41598-022-22128-w)
Supplement: Supplementary file 1 — Supplementary Information. [file 41598_2022_22128_MOESM1_ESM.docx]

Supplementary information for

**Subcutaneously implantable electromagnetic biosensor system for continuous glucose monitoring**

*Seongmun Kim, Jagannath Malik, Jong Mo Seo, Young Min Cho and Franklin Bien**

Method 1. Electromagnetic sensor

- Section.1: Sensor modelling and near *-field* simulation
- Section.2: In vitro sensor test with aqueous glucose solution
- Section.3: Phantom test as artificial bio-environment

Method 2. Long-term OGTT with Interface board

- Section.4: OGTT in beagle long-term test setup

Method 3. Data processing

- Section. 5: Noise filtering using predictive Kalman filtering
- Section. 6: Method of linear regression
- Section. 7: Mean absolute relative difference (MARD) analysis

Figure

- Fig. S1. Simulation model of the proposed sensor (a, b)
- Fig. S2. In vitro measurement of permittivity and sensor frequency characteristics (a–e)
- Fig. S3. Phantom environment and results. (a-c)
- Fig. S4. Surgery for inserting the sensor under the skin and sensor interface board (a-d)
- Fig. S5. Kalman filter application case for noise removal and trend finding. (a-c)

Table S1

- Phantom ingredients for bio environment condition

Reference List

Method 1. Electromagnetic sensor

**Section.1: Sensor modelling and near*-field* simulation**

Three types of field regions surrounding an electromagnetic resonator/antenna are associated with radiating energy and reactive energy. The field regions are the reactive near-field region, radiating near-field region (Fresnel zone), and far field region (Fraunhofer zone). The proposed sensor is non-radiating in nature and has a strong near field. This enhances the dielectric sensing performance with minimum radiation of energy fed to the sensor.


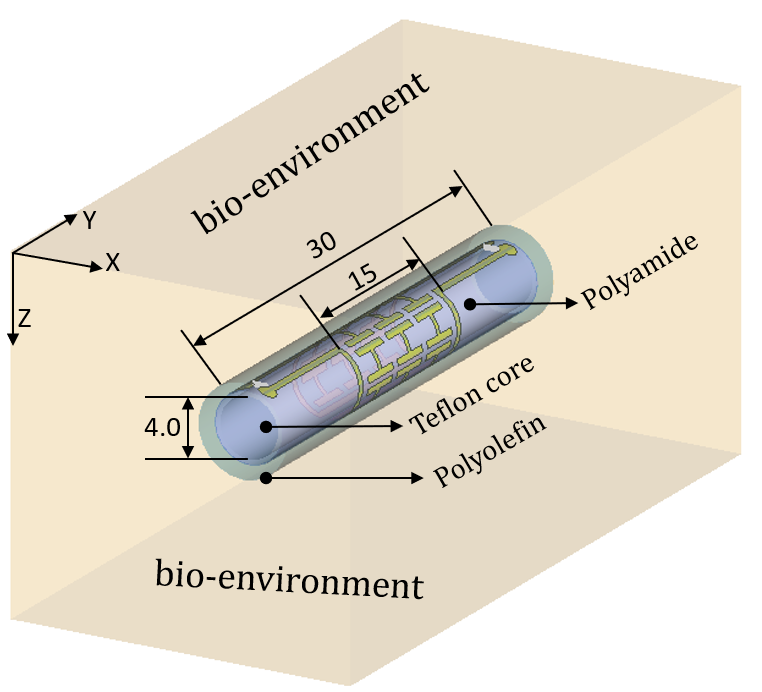

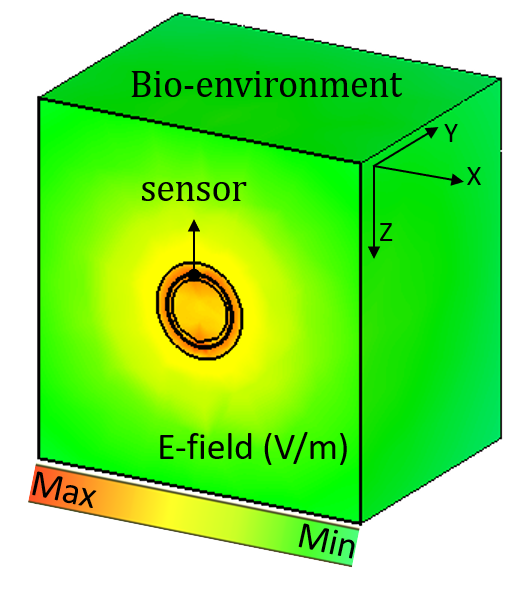


(a) (b)

**Figure S1** Simulation model of the proposed sensor (*not to the scale, unit: mm*). (a) Sensor with *Teflon* inner core and *Polyolefin* cover embedded in bio-environment model in CST simulator (b) simulated surrounding near-field of the sensor.

The working principle of the proposed sensor is to detect minute permittivity changes of the surrounding material. This permittivity changes are reflected in sensor resonance frequency. Hence, we defined *sensitivity* of the sensor as the amount of resonance frequency shift owing to small permittivity changes of bio-environment. Modelling and simulation of the sensor is done using FDTD based full wave simulator CST microwave studio (Fig. S1(a)). Transient mode simulation is used to check sensor resonance and near field characteristics. We have considered dispersive bio-environment model defined in the simulator. A series of parametric optimization was done to maximize sensor *sensitivity*. The sensor can be excited at either of the two ports while measuring the sensor resonance at the other port. It can also be operated as a single port or dual port system while measuring the reflection parameter at the ports. The sensor has a Teflon inner core and a polyolefin cover isolates the sensor from the direct contact with surrounding. Without this cover, the presence of water at implant site can cause electrical shorting of sensor terminals. This cover also affects sensor sensing capabilities. Cover having low dielectric permittivity and loses with thinner thickness is suitable for present case. Simulated near electric field strength is shown in Fig. S1(b) on a planar cross section normal to the antenna surface. It can be seen that the electric field are strongly distributed closely to the sensor surface. Electric field strength decays rapidly away from the sensor. This locally distributed field is responsible for the sensing ability of the sensor. The resonance frequency of the sensor follows an inversely proportional relation with the permittivity variations. The sensor is intended for subcutaneous implantation. It is sensitive to few mm (~ 5 mm) from sensor surface surrounding uniformly around the sensor.

Previously published research data indicated that the permittivity of the blood plasma changes with the changing glucose level over a wide frequency range. This frequency-dependent complex permittivity variation with the BG level fits in the Debye dispersion model. The variation in the permittivity is higher as we move to a higher frequency; however, the system on chip (SoC) device was extremely difficult to implement. The lower frequency spectrum was limited mainly due to the practical size of the sensor required. Considering these facts, the focus was to develop an electromagnetic (EM) sensor about L- or S- band, more specifically around the ISM band.

The local specific absorption rate (SAR) is more meaningful in our case compared to the whole-body average SAR. Typical local SAR values are averaged in tissue masses of approximately 10 g, as specified by the Telecommunication Technology Council Agenda No. 89 and CENELEC 1995, whereas a value of 1 g is adopted by ANSI/IEEE C95.1-1992 of the United States. SAR is calculated from the total power absorbed in a biological tissue and other lossy material. This does not consider the power loss in lossy metals. It can be expressed as:

$SAR=\left( \frac{\sigma{.\left| E \right|}^{2}}{\rho} \right)$ . (1)

Where, *E*: rms value of the electric field strength in the tissue in V/m

σ: conductivity of body tissue in S/m; ρ: density of body tissue in kg. m^-3^

The SAR value is expressed in units of watts per kilogram (W. kg ^-1^). The power loss density (PLD) value is expressed in units of watts per cubic meter (W. m ^-3^). It is calculated using equation (1). The proper scaling to a user-defined accepted power requires the balance of excitation. Choosing AR filter results, calculations of the excited and accepted power by the EM resonator can be improved. If the SAR result has been generated by a circuit simulation, system power values are preferable. However, it is not required at this stage. It is necessary when using an SoC with an implant sensor. IEEE/IEC 62704-1 is the standard averaging method, and it was adopted to calculate SAR in the present case. The proposed sensor can be operated with low power, i.e., 0 dBm (1 milliwatt) of power, to function properly. However, due to possible attenuation/loss in packaging material, slightly higher power can be considered while keeping SAR within a safe limit. In the simulation, with an excitation of 10 dBm RF power level, the maximum SAR for the proposed sensor is ~ 0.44 W/kg (1-g average), which was obtained from the full wave simulation results in CST Microwave Studio. During the animal experiment, the excitation power in the VNA was set to 0 dBm. This ensured a very good safety measure, and the sensor still showed good sensitivity in the animal experiment with a low input power. The safety limits for SAR in the U.S.A and Council of the European Union are 1.6 W/Kg (1-g tissue averaged) and 2.0 W/Kg (10-g tissue averaged), respectively. The present sensor simulation result is significantly lower than both regulations.

**Section.2: In vitro sensor test with aqueous glucose solution**

To verify sensor sensitivity towards permittivity changes, in vitro experiments were done with aqueous glucose solution with varying glucose concentration. The dielectric permittivity of glucose solutions was measured using Keysight performance probe. Sensor resonance frequency were also measured to understand sensor ability to detect glucose dependent small permittivity changes of different solutions (Fig. S2a). Hence, glucose solutions were prepared by adding appropriate amount (using a weighing scale) of D-glucose (Sigma-Aldrich) in DI water. Five different samples (100, 200, 300, 400, 500 mg/dL) were prepared. After preparing solutions, it was stirred continuously for 30 minutes using a magnetic stirrer inside sealed container. To make a homogeneous glucose concentration and permittivity distribution, solutions were kept in a temperature-controlled chamber for overnight. We measured the permittivity of the solutions using a performance probe (N1501A, Keysight) after calibrating the probe. Precautions were taken during measurement to reduce noise in the measurement, e.g., fixing the cable so that it does not move or twist after calibration, removing air bubbles from probe tip, maintain all solutions at same temperature etc. From the in vitro permittivity measurement (in a temperature-controlled environment) of aqueous glucose solutions, it was observed the permittivity (real part) decreases with increase in the glucose level in the solution (Fig. S2b). However, complex part of the permittivity does not show any kind of variation with different glucose concentration (Fig. S2c). The trend was consistent with other previous studies. The next step was to check sensor ability to detect this permittivity variations due to different glucose level.


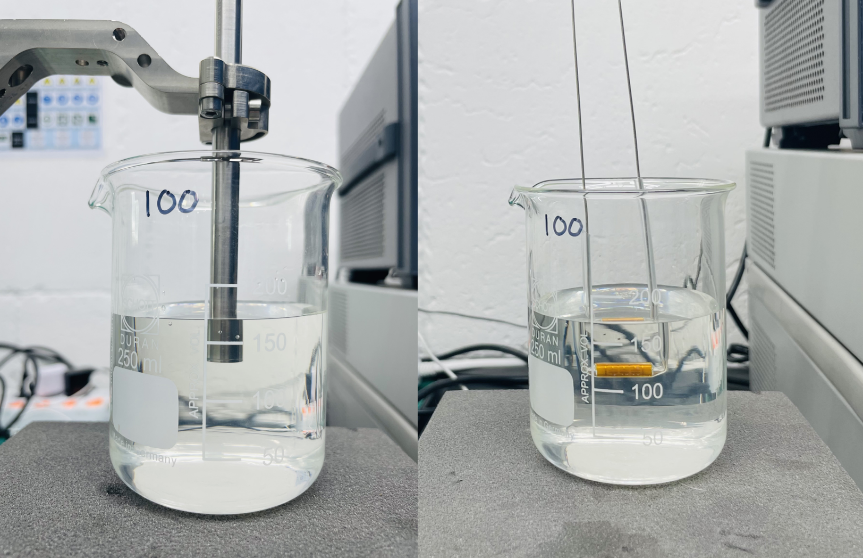


(a)


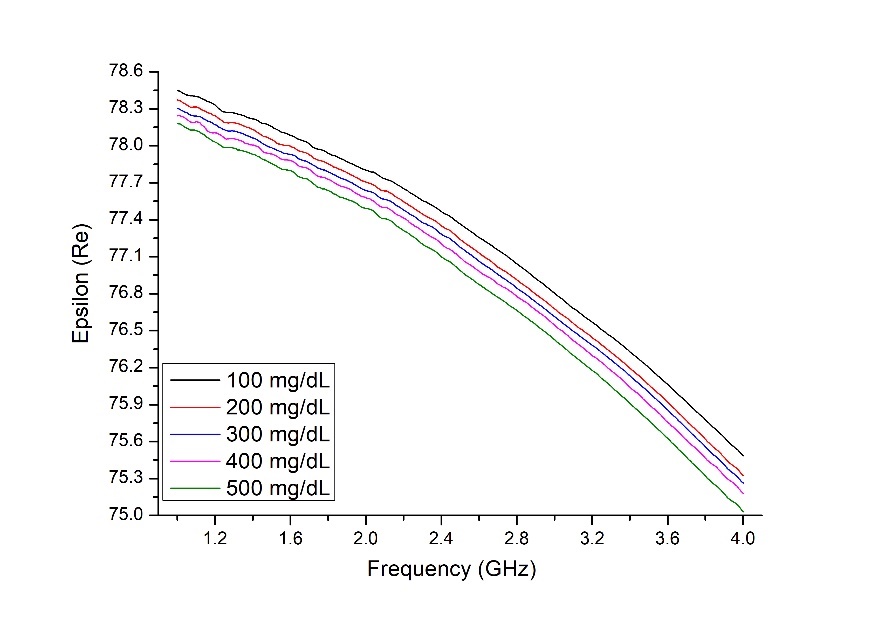


(b)


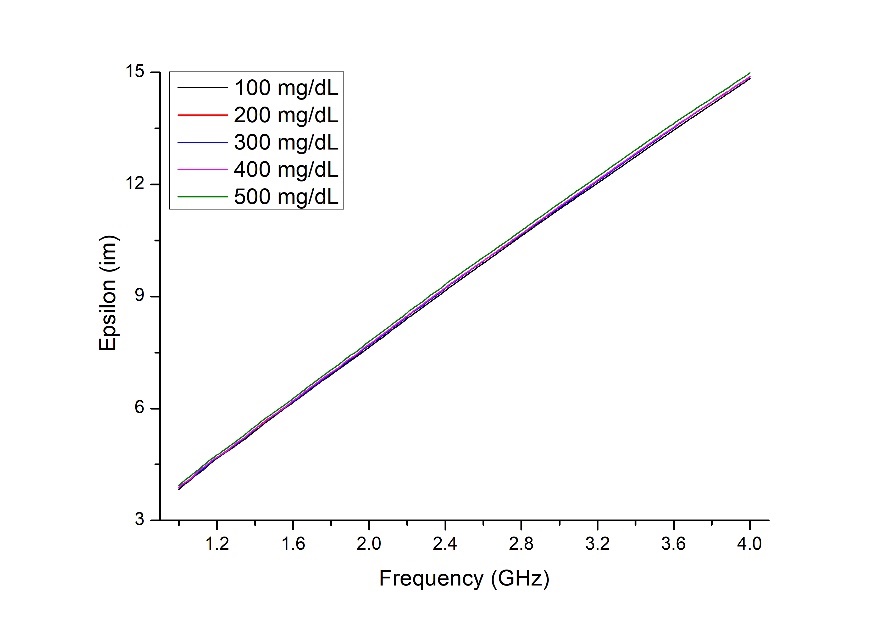


(c)


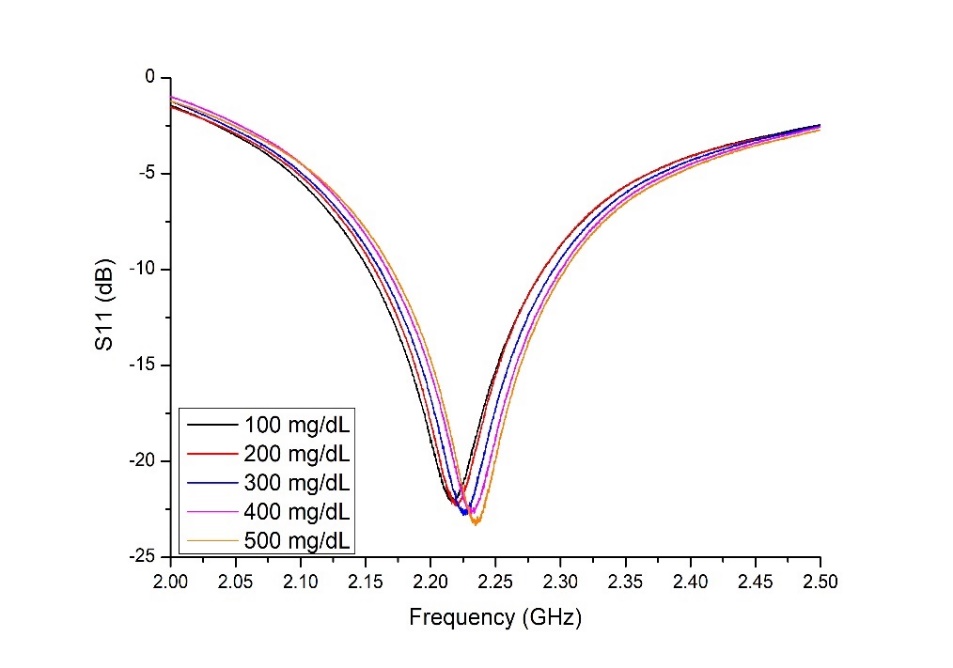


(d)





(e)

**Figure S2** In vitro measurement of permittivity and sensor frequency characteristics. (a) Experimental setup for measuring permittivity of glucose solution and sensor resonance frequency (b) permittivity real part (c) permittivity imaginary part (d) sensor frequency according to glucose level in aqueous solution (e) continuous measurement of sensor frequency with changing temperature of DI-water

The sensor was fabricated using flexible polyamide substrate (0.15 mm thickness) with 35 µm of copper thickness. The reason for choosing flexible substrate is to make the sensor cylindrical form by warping it around a Teflon core as modelled in the simulation. Two semi-rigid coaxial cables were soldered to the 2-ports of the sensor. We ensured the section of cables soldered to the sensor was properly sealed and does not contact directly with the solution. For the same, polyolefin tubes were used to cover the sensor entirely and soldered portions of coaxial cables at both ends. Cables lengths were considered exactly during calibration of network analyzer for de-embedding. The measured scattering parameters were symmetric for both ports. Sensor resonance frequency was measured with the solutions by dipping the sensor into the solution. Each time the sensor was dipped into one solution and resonance frequency saved. After removing sensor from the solution, it was wiped, dried before measuring another solution. It was observed sensor resonance frequency increased with increasing glucose level in the solution. Sensor frequency changed about 26 MHz for glucose concentration changing from 100 mg/dL to 500 mg/dL in DI water solution condition (Fig. S2d).

The sensor frequency shifting with continuous permittivity changes has been evaluated in laboratory experiment. Permittivity of water changes with temperature variations. Increasing the temperature, permittivity of water decreases. Considering this temperature dependent property of water, an experimental environment was setup to messure sensor performance in continuous permittivity varying medium. Accordingly, the sensor was dipped in side water along with a temperature sensor in a temperature-controlled water bathtub (*C-WBD3, Changshin Science, Korea*). The water temperature can be raised quickly to the user set temperature level. The temperature sensor (*34308-60001 precision thermistor, Keysight*) was connected to a digital multimeter (*34461A, Keysight*) to read real-time temperature of water. The digital multimeter and network analyzer were connected to a PC running automation program over LAN interface. The measurement data were recorded using an in-house developed automation program. From Fig. S2e, it can be seen that the sensor has no delay and quickly responds to the dielectric changes. With increasing temperature, permittivity of water decreases and sensor frequency increases. After reaching the maximum temperature, the permittivity changes stopped, also variation in sensor frequency. This confirms the proposed senor is suitable for continuous permittivity tracking.

**Section.3: Phantom test as artificial bio-environment**

A phantom model mimicking a bio-environment was prepared in the laboratory to determine the resonance stability of the sensor when it is inserted into a living body. The materials used for manufacturing the phantom are specified in Supplementary Table 1. The texture, stiffness or elasticity, and dielectric permittivity (fat or muscle) can be adjusted by suitably changing the ratio of materials while preparing the phantom. A phantom dielectric permittivity (around 50) similar to the muscle tissue was prepared and verified using (N1501A Performance Probe, Keysight, USA) a 2–3 GHz sensor operating frequency band. Polyethylene powder can control the dielectric constant value such that it is similar to that of the muscle and skin tissue with appropriate mixing ratio. TX-151 is a material that can hold and maintain the shape of the phantom. It also adjusts the shape and adheres to the sensor as much as possible over time^1,2^.

Initial measurements of sensor resonance variations with changing environment condition and temperature were performed to tune the sensor before the actual in vivo test. In the actual in vivo implant case, the cells and tissues may gather around the sensor, and the surface contact changes over time after sensor insertion into the body. This may affect the sensor performance or shift the frequency to another band. It is also important to ensure that the sensor produces a stable resonance frequency and sensitivity towards dielectric permittivity changes and does not get affected by small environmental changes.

To verify this, we conducted a four-day-long experimental data recording of sensor frequency after inserting it into the phantom sample. We also recorded the temperature data using a miniature temperature sensor (34308-60001-precision thermistor, Keysight, USA) inserted into the phantom together with the proposed sensor. The thermistor was connected to a digital multimeter (34461A, Keysight, USA) to record the real-time temperature. All resonance frequency data and temperature data were recorded using a Windows automation program. Fig. S3a and Fig. S3b. shows the measurement environments for the stable sensor data analysis while inside the phantom. The temperature variation of the middle part of the phantom, where the sensor is inserted, is $23\pm0.25 ℃$, which is slightly lower than the outside temperature. The results (Fig. S3c upper graph) show the resonance frequency and temperature variations over four days. Gradually, the phantom surrounds the sensor, and the resonance frequency changes according to the contact surface. Approximately 3 MHz of sensor frequency variation was observed over four days. This ensures sensor stability for long-term applications. The internal temperature variations also do not affect the sensor frequency. The change in the internal temperature of the phantom is approximately 0.5, and result (Fig. S3c lower) shows that it can be seen that it can be maintained at $23℃$. The frequency variation due to the change in the surface contact is much smaller than the frequency variation observed in the actual IVGTT in swine and beagle.


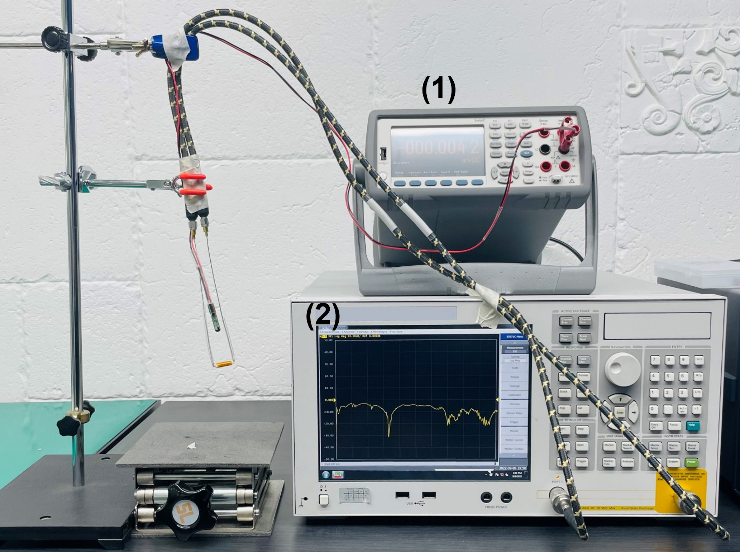

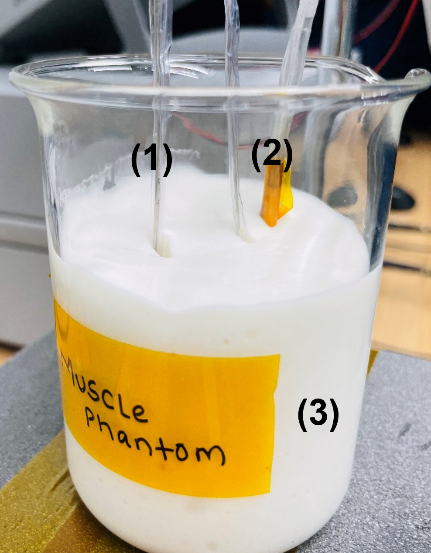


(a) (b)

**
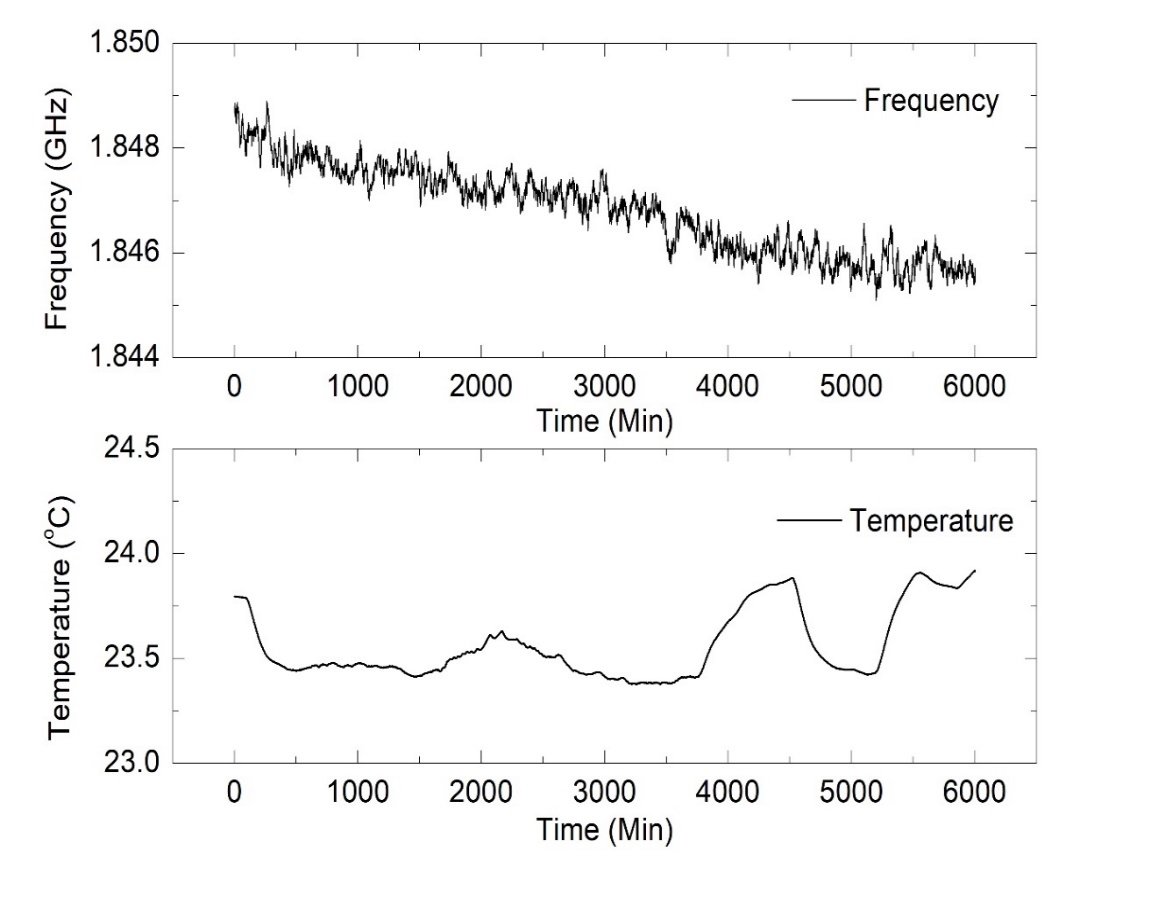
**

(c)

**Figure** S3**. Phantom environment and results.** **a,** The measurement environment: (1) DMM (Digital Multimeter), (2) VNA (Vector Network Analyzer). **b,** the measurement bio -environment: (1) Proposed sensor based on EM. (2) Temperature sensor. (3) Phantom model.

**c,** sensor frequency drift (1.849 GHz to 1.846 GHz) over four days inside phantom, maximum temperature variation inside phantom was only ~ $0.5℃$ .

**Method 2. Long-term OGTT with Interface board**

**Section.4: OGTT in beagle long-term test setup**

For long-term evaluation of the proposed sensor, we used an in-house developed sensing interface board (circuit module and mobile application) for transmission and reception of the sensor data. We developed an android application to control the sensor and display data in real time. The mobile application can control the frequency sweep and the resolution bandwidth of the RF generator realized using a phase-locked loop (PLL). The board is made up of three sub parts that can perform individual roles. The power management part is composed of voltage regulator and low drop output (LDO) to supply stable power with reduced noise. The interface board is powered up using an external battery (10,000 mAh, *Joy room, China*). Through the received power, the Communication and Control part exchanges data with the external mobile through a micro controller unit (MCU).

The PLL generates an RF signal that passes through a coupler to the sensor. The forward power and reflected power from the sensor are used to calculate the resonance frequency. The PLL operates over a frequency band of 1.7–2.7 GHz with a sweeping resolution of 1 MHz per step. It can be operated at an integer mode and a fractional mode. The integer mode locates the resonance from a wide frequency sweep at the 1 MHz step. The fractional mode which is one of the methods for generating frequency on PLL has fine search around the resonance dip with a 10 kHz step for more accurate information. After the integer mode locates the resonance point, for a more accurate measurement, the fractional mode operates over a 10 MHz bandwidth on either side of the resonance point. A dual scan mode is used to fractional and integer mode.

A coupler is used for separating signals (reference signal, reflected signal and signal toward to sensor). the coupler has electric characteristic of isolation and pass on signal.

A signal can only pass in one direction from a specific port to a port through the coupler. Conversely, when a signal flows, the isolation characteristic of the coupler prevents the signal from passing through. Each signal which is separated by coupler is flowed to enveloped detectors Envelope detectors are used to convert the reflected signal ($S_{11}$) and transmitted signal ($S_{21}$) to a lower DC level before feeding it to the analog digital convertor (ADC). The converted digital data are transmitted to the mobile through Bluetooth. The mobile application stores data and gives a plot of real-time resonance behavior of the implant sensor. For long-term test, proposed sensor was inserted with interface board on beagle. Long-term environment was in figure S4.


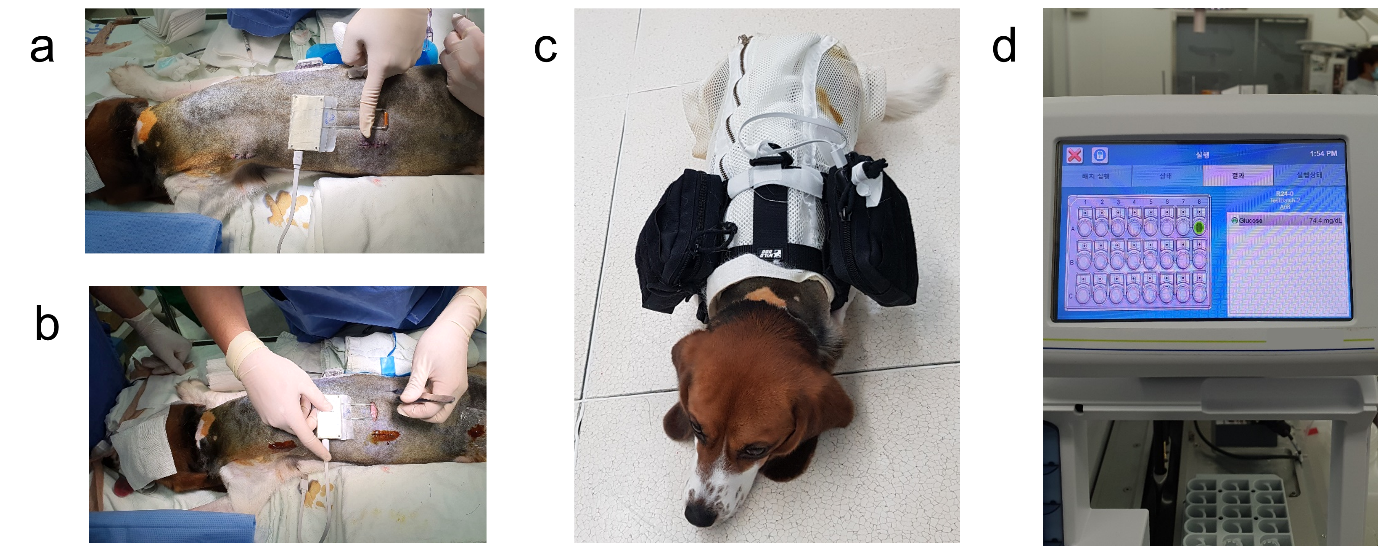


**Figure** S4**. Surgery for inserting the sensor under the skin and sensor interface board.** (**a)** sensor insertion position, (**b)** Interface board and sensor (**c)** portable battery in a vest (**d)** reference BGL measuring equipment

**Method 3. Data processing**

**Section. 5: Noise filtering using predictive Kalman filtering**

The continuous measurement data from the implant sensor were stored at each second. Practically, there are many noises present in the raw data. Potential noise sources corrupting the sensor data arise from the instrumental noise, small sensor movement due to the animal breathing rate, and high-frequency noise inherent in the RF. Therefore, it is essential to remove noise and retrieve clean sensor data. It is also important to clean the data such that small changes in BG and the corresponding sensor frequency changes can be distinguished with high reliability.

One of the methods to remove the measurement error, distortion, and white noise components is to use the numerical filtering algorithm. Linear averaging is the simplest algorithm that operates like a low-pass filter and removes high-frequency noises. It is mostly suitable for processes that involve slow variations. However, it may also cause loss of information.

Linear filtering is suitable in most cases owing to its computationally efficient implementation. Kalman filter, which is used extensively in the industrial field, can effectively remove noise. The Kalman filter is a recursive filter that estimates an accurate state space (predict and update) from noisy measurements. The algorithm is modified according to our problem to define the state variables and observable variables^3,4^. The expression of the implemented Kalman filter is as follows:

$\hat{x}_{k}^{-}=F\hat{x}_{k-1}^{+}$ (2)

$F=\left[ \begin{matrix} 1 \\ \Delta t \\ \frac{1}{2}\Delta t^{2} \end{matrix} \right]$ (3)

$Q=\left[ \begin{matrix} \frac{1}{2}\Delta t^{2} \\ \Delta t \\ 1 \end{matrix} \right]\times\left[ \begin{matrix} \frac{1}{2}\Delta t^{2} \\ \Delta t \\ 1 \end{matrix} \right]^{T}$ (4)

$\hat{x}_{k}=F\hat{x}_{k-1}+FP_{k-1}H^{T}(R+HP_{k-1}^{+}H^{T})^{-1}(Z_{k}-H\hat{x}_{k-1})$ (5)

$P_{k}^{+}=FP_{k-1}^{+}F^{T}+Q-FP_{k-1}^{+}H^{T}(R+HP_{k-1}^{+}H^{T})^{-1}HP_{k-1}^{+}F$ (6)

Where, $\hat{\left( . \right)}$ denotes the estimation parameter and ${(.)}^{T}$ denotes the matrix transpose. $\hat{x}_{k}^{-}$ is the state estimation before correction at time step $k$, and $\hat{x}_{k-1}^{+}$ is the state estimation corrected at time step $k-1$ in the equation (2). $P_{k}^{-}$ is in the form of a state error covariance matrix before correction at time k. $P_{k}^{-}$ can be defined as a state error covariance matrix corrected at time k-1. $F$ is a state transition matrix, and it is defined as a value for time based on the expression of position movement. Assume that $\Delta t$ is the time constant at which system updates in equation (3). H is a measurement matrix. $\hat{x}_{k}$ is the current state, and $z_{k}$ is the frequency data of the sensor. The noise component is set to $Q$ and $R$. $Q$ is a factor parameter and indicated the amount of change in $F$ in equation (4). The noise component is included in the present state equation (5) and updated error covariance equation (6). Finally, we determine the trend of the measured data while removing noise with the values of $z_{k}$, $F$ and $R$. Fig. S5a and Fig. S5b are the test result of the Kalman filter applied to the raw noisy data for the swine and beagle, respectively. It can be observed that the frequency data before the noise correction has spikes and high-frequency variations. The filtered data has a clear trend depending on the BG level changes. The higher the value of the time parameter $\Delta t$, the more it follows the original data; and the lower the value, the smoother the data slope. This parameter controls the sampling rate in algorithm between the two observations.





(a)





(b)





(c)

**Figure S5. Kalman filter for noise filtering and trend finding. a,** Swine result graph ($R=0.005$, $\Delta t=0.05$). **b,** Beagle result graph ($R=0.005$, $\Delta t=0.05$). c, Filtering performance: Raw data, Kalman filtering and moving average filtering with window size 10, 50, 100

Another possible filtering method is to implement moving average filter to remove the noise and get a clear trend in time series data. The smoothness in filtered data highly depends on the window size. A larger window gives a smoother trend however also needs more memory to store previous time series data for filter implementation. It also adds delay factor in the filter data trend depending on the window size compared to original trend (Fig. S5 c). Results for window size 10,50,100 are shown for comparison. In the case of Kalman filtering, it requires only one previous time step data for implementation. Results of Kalman filtering also shown in Fig. S5c and compared with moving average filtering.

**Section. 6: Method of linear regression**

It was observed that the dielectric permittivity is inversely proportional to the BG level. With the increasing glucose concentration, the dielectric constant decreases, and the sensor resonance frequency shifts to a higher frequency. Therefore, a positive correlation exists between the sensor frequency and the BG level. We adopted a regression model to show the linear relationship between the BG level and the resonant frequency^5^.

$A=\overline{Y}-b\overline{X}$ , (7)

$b=r(X,Y)\frac{S_{y}}{S_{x}}$ , (8)

$Y^{'}=bX+A$ , (9)

where X is BG level and Y is resonance frequency. $r(X,Y)$ is the correlation coefficient between X and Y. The relationship between the two variables can be fitted to a linear relationship with minimal system error averaged over all data points. $S_{x}$ and $S_{y}$ are the standard deviation of BG level and sensor frequency, respectively. $\overline{X}$ and $\overline{Y}$ are the means of the BG level and sensor frequency, respectively. A linear relationship can be obtained by setting the measured BG level and sensor data. When applied to equation (9), the linear correction coefficient ($R^{2}$) can be calculated. The linear correlation coefficient can be derived using equation (10).

$R=\frac{\sum(x-m_{x})(y-m_{y)}}{\sqrt{\sum{(x-m_{x})}^{2}\sum{(y-m_{y})}^{2}}}$ (10)

Using the regression equation established between the BG level and sensor frequency, the predicted BG level can be obtained from the corresponding sensor frequency data. Similarly, a change in the BG level can be determined from the sensor frequency deviation.

**Section. 7: Mean absolute relative difference (MARD) analysis:**

MARD is an indication of error rate scale by comparing measured BG level (obtained from sensor) and the reference BG level (YSI) value. As per the commercial BG measurement meters, the standard error rate falls within 15%. The reference BG level is measured using YSI equipment which is gold standard to analyze glucose and lactate components in a sample. It is a reference equipment for most BG measurement methods^6^. The level of MARD is calculated by comparing the measured BG level and the reference BG level^7^. In addition, MAD can be used to calculate the overall error average using equations (11) and equations (12) respectively.

$MARD=\frac{1}{n}\sum_{i=1}^{n} |\frac{\mathrm{BG}_{\mathrm{predicted}}(i)-\mathrm{BG}_{\mathrm{Ref}}(i)}{\mathrm{BG}_{\mathrm{Ref}}(i)}|$ (11)

$MAD=\frac{1}{n}\sum_{i=1}^{n} |\mathrm{BG}_{\mathrm{predicted}}(i)-\mathrm{BG}_{\mathrm{Ref}}(i)|$ (12)

$\mathrm{BG}_{predicted(i)}$ is the measured glucose concentration value that is converted from frequency to glucose level. That value is derived through linear regression. $\mathrm{BG}_{Ref(i)}$ is measured using YSI2500 (Yellow Springs Instruments, Yellow Springs, OH).$n$ represents the number of measurements. MARD is used to obtain the ratio of the difference between $\mathrm{BG}_{predicted(i)}$and $\mathrm{BG}_{\mathrm{Ref}}(i)$. MAD is used to confirm the absolute mean error between the measured value and the reference value. Through the numerical value and distribution of MAD, it is possible to identify the band in which the comparison value differs the most.

**Table S1. Phantom ingredients for bio environment condition**

| Materials | Weight Ratio [%] |
| --- | --- |
| DI-Water | 81.5 |
| $\mathrm{Na}N_{3}$ | 0.05 |
| TX-151 | 2 |
| NaCl | 0.6 |
| Polyethylene Powder | 13.3 |
| Agar | 2.5 |

**Reference**

1. Onishi, T., & Uebayashi, S., biologicla tissue-equivalent phantoms usable in broadband frequency range, NTT DoCoMo Tech. 7, 4, 61-65 (2006).
2. Bonds, Q., & Weller, T., Multi-layer RF tissue phantoms for mimicking a human’s core, Conf. Microw. Antennas Commun. Electron. Syst. COMCAS, 2017, pp. 1-4 (2017).
3. Knobbe, E. J., & Buckingham, B., The Extended Kalman Filter for Continuous Glucose Monitoring, Diabetes Technol. Ther. 7, 1, 15-27 (2005).
4. Bequette, B. W, J., Continuous Glucose Monitoring Real-Time Algorithms for Calibration, Filtering, and Alarms, Diabetes Sci. Technol. 4, 2, 404-418 (2010).
5. Zanon, M., et al., Assessment of Linear Regression Techniques for Modeling Multisensor Data for Non-Invasive Continuous Glucose Monitoring, Annu. Int. Conf. IEEE Eng. Med. Biol. - Proc. 2538–2541 (2011).
6. DeSalvo, D. J., Shanmugham, S., Ly, T. T., Wilson, D. M., & Buckingham, B. A, J., Accuracy Evaluation of Blood Glucose Monitoring Systems in children on overnight Closed Loop Control, Diabetes Sci. Technol. 8.5: 969-973 (2014).
7. Jain, P., Maddila, R., & Joshi, A. M., A precise non‑invasive blood glucose measurement system using NIR spectroscopy and Huber’s regression model, Opt. Quantum Electron. 51, 2, 51(2019).

**Abbreviation List**

BG, Blood Glucose

RF, Radio Frequency

PLL, Phase-Locked Loop

ADC, Analog-to-Digital Convertor

MCU, Micro Controller Unit

LDO, Low Drop Output

GTT, Glucose Tolerance Test

IVGTT, Intravenous Glucose Tolerance Test

OGTT, Oral Glucose Tolerance Test

EM, Electromagnetic

VNA, Vector Network Analyzer

MARD, Mean Absolute Relative Difference

MAD, Mean Absolute Difference

EGA, Clarke Error Grid Analysis

PEP, Polyethylene powder

SAR, Specific absorption rate
